# Serious Adverse Events and Laboratory Monitoring Regimens for Outpatient Parenteral Antimicrobial Therapy With Cefazolin and Ceftriaxone

**DOI:** 10.1093/ofid/ofad606

**Published:** 2023-12-02

**Authors:** Shawnalyn W Sunagawa, Sarah Arduser, Molly M Miller, Elizabeth Lyden, Melissa LeMaster, Nicolas Cortes-Penfield, Richard J Hankins, Scott J Bergman, Bryan T Alexander

**Affiliations:** Department of Pharmaceutical and Nutrition Care, Nebraska Medicine, Omaha, Nebraska, USA; College of Pharmacy, University of Nebraska Medical Center, Omaha, Nebraska, USA; Department of Pharmaceutical and Nutrition Care, Nebraska Medicine, Omaha, Nebraska, USA; College of Pharmacy, University of Nebraska Medical Center, Omaha, Nebraska, USA; Department of Pharmaceutical and Nutrition Care, Nebraska Medicine, Omaha, Nebraska, USA; College of Public Health, University of Nebraska Medical Center, Omaha, Nebraska, USA; Department of Pharmaceutical and Nutrition Care, Nebraska Medicine, Omaha, Nebraska, USA; Divison of Infectious Diseases, University of Nebraska Medical Center, Omaha, Nebraska, USA; Divison of Infectious Diseases, University of Nebraska Medical Center, Omaha, Nebraska, USA; Department of Pharmaceutical and Nutrition Care, Nebraska Medicine, Omaha, Nebraska, USA; College of Pharmacy, University of Nebraska Medical Center, Omaha, Nebraska, USA; Department of Pharmaceutical and Nutrition Care, Nebraska Medicine, Omaha, Nebraska, USA

**Keywords:** adverse events, cefazolin, ceftriaxone, laboratory techniques and procedures, outpatient parenternal antimicrobial therapy

## Abstract

The optimal laboratory monitoring frequency for outpatient parenteral antimicrobial therapy–related adverse events (OPAT-AEs) during cefazolin and ceftriaxone therapy is not well defined. We identified 2.7 OPAT-AEs per 1000 sets of weekly laboratory tests in this population, suggesting that less intensive laboratory monitoring may be safe and reasonable.

Outpatient parenteral antimicrobial therapy (OPAT) facilitates earlier hospital discharge, reduces health care costs, and enhances patient comfort [1–3]. There is little high-quality evidence to support specific laboratory test frequencies for monitoring individual antimicrobials utilized in OPAT [3, 4]. Indeed, while the 2018 Infectious Diseases Society of America (IDSA) clinical guideline recommends frequent (often weekly) laboratory testing in patients receiving OPAT, its authors found no published studies directly addressing which or how often labs should be ordered.

Prior studies suggest that OPAT-related adverse events (OPAT-AEs) occur in ∼20% of patients, with ∼15% of these being clinically significant. However, definitions of what constitutes a clinically significant adverse event (AE) vary [5–9]. Additionally, these studies grouped different antimicrobials and included agents such as aminoglycosides and vancomycin, which have less favorable AE profiles than beta-lactams. Zukauckas et al. reviewed OPAT patients prescribed beta-lactams and found that while 52% had abnormal laboratory findings, only 5.7% of these abnormal laboratory findings led to an antimicrobial therapy modification [8]. Beyond this work and a recent retrospective cohort study assessing laboratory monitoring for all antibiotics administered via OPAT [10], little is known about the cadence of laboratory monitoring necessary to prevent AEs in patients receiving beta-lactams via OPAT. As cefazolin and ceftriaxone comprise a large percentage of overall OPAT utilization, more rigorous and directed evaluation of the necessary monitoring for these agents would be valuable.

The Nebraska Medicine OPAT program is a multidisciplinary team of infectious diseases clinicians who evaluate (via electronic consultation) inpatients for whom outpatient intravenous antimicrobials are requested, then follow those patients during OPAT. Our screening process helps ensure that patients are discharged with the most appropriate antimicrobial care plan and assists with monitoring for OPAT-AEs. The aim of this study was to assess OPAT-AE rates for patients receiving cefazolin and ceftriaxone to better define appropriate OPAT laboratory monitoring for these antimicrobials.

## METHODS

We retrospectively evaluated patients prescribed either cefazolin or ceftriaxone via OPAT between March 1, 2019, and September 30, 2022, and followed by our OPAT program. Patients receiving multiple intravenous antimicrobials were excluded; however, patients receiving concomitant oral antibiotics were included.

The primary outcome was incidence of clinically significant OPAT-AEs, defined as drug-associated AEs (eg, abnormal labs, allergic reactions, *Clostridioides difficile* infection) leading to a change in antimicrobial therapy or any catheter-associated AE requiring intervention. Secondary outcomes included time from start of antimicrobial therapy to clinically significant AEs, unplanned health care utilization (eg, emergency department visits, readmissions), and OPAT actions performed to obtain or clarify laboratory monitoring data. Our institutional review board deemed this a quality improvement project exempt from review.

Each OPAT-AE was independently adjudicated by 2 study investigators. We performed descriptive statistics as well as Fisher exact and independent-sample *t* tests to assess univariate associations. Multivariable logistic regression with backward selection was used to determine if variables significant on univariate analysis remained independently predictive of OPAT-AEs after adjusting for other covariates in the model. All analysis was done using SAS, version 9.4 (SAS Institute Inc.), and *P* values <.05 were considered statistically significant.

## RESULTS

Our study included 708 courses of OPAT comprising 22 975 days of therapy. Patients in our cohort received a total of 3282 sets of weekly labs (typically including a complete blood count with differential and serum chemistries, with or without liver function tests), of which 99.7% were reviewed within 72 hours from lab draw; 263/708 OPAT courses (37.1%) required OPAT team actions to obtain missing or appropriate labs for review on at least 1 occasion. Of the 708 courses, 366 (51.7%) utilized cefazolin, and the median total duration of therapy (interquartile range [IQR]) was 31 (19–42) days. Most patients completed OPAT via home health care (58.6%), and the most common indication for OPAT was osteomyelitis (17.2%). Additional cohort characteristics are listed in Table 1.

**Table 1.** OPAT Cohort Characteristics

Characteristics (n = 708)^a^	No. (%)
Male	418 (59)
Age, mean ± SD, y	59.1 ± 16.3
Weight, mean ± SD, kg	91.2 ± 27.9
Charlson Comorbidity Index score, median (IQR)	4 (2–7)
Immunocompromised^b^	191 (27)
Antimicrobial agent	…
Cefazolin	366 (51.7)
Ceftriaxone	342 (48.3)
Concomitant oral antimicrobial therapy	111 (15.7)
Metronidazole	53 (7.5)
Rifampin	36 (5.1)
Doxycycline	6 (0.8)
Fluconazole	4 (0.6)
Fluconazole and metronidazole	3 (0.4)
Doxycycline and metronidazole	2 (0.3)
Amoxicillin/clavulanate	1 (0.1)
Clindamycin	1 (0.1)
Itraconazole	1 (0.1)
Linezolid	1 (0.1)
Trimethoprim/sulfamethoxazole	1 (0.1)
Trimethoprim/sulfamethoxazole and metronidazole	1 (0.1)
Vancomycin	1 (0.1)
Total duration of therapy, median (IQR), d	31 (19–42)
Days of therapy on drug of interest, median (IQR)	30 (19–42)
OPAT modality	…
Home health care	415 (58.6)
Skilled nursing facility	135 (19.1)
Long-term acute care	57 (8.1)
Hemodialysis center	51 (7.2)
Infusion center	49 (6.9)
Other	1 (0.1)
Vascular access	…
PICC/CVC	589 (83.2)
HD access	59 (8.3)
Midline	53 (7.5)
PIV	7 (1)
OPAT indications	…
Osteomyelitis	122 (17.2)
Prosthetic joint infection	120 (16.9)
Primary/catheter-related bloodstream infections	111 (15.7)
Infective endocarditis/vascular or cardiac device infection	89 (12.6)
Skin and soft tissue/trauma/wound infection	79 (11.1)
Septic arthritis	53 (7.5)
Intra-abdominal infection	39 (5.5)
Central nervous system/neurologic infection	39 (5.5)
Lung abscess/empyema/pneumonia	31 (4.4)
Urological infection	16 (2.3)
Other	9 (1.3)
Pathogens isolated	…
*Staphylococcus aureus*	291 (41.1)
*Streptococcus* spp.	155 (21.9)
Polymicrobial	61 (8.6)
None identified	59 (8.3)
Coagulase-negative *Staphylococcus* spp.	41 (5.8)
*Escherichia coli*	26 (3.7)
*Cutibacterium acnes*	19 (2.7)
Other	56 (7.9)

Abbreviations: HD, hemodialysis; IQR, interquartile range; OPAT, outpatient parenteral antimicrobial therapy; PICC/CVC, peripherally inserted central catheter/central venous catheter; PIV, peripheral intravenous catheter.

^a^Six hundred sixty-six unique patients, 38 patients with >1 OPAT course of therapy.

^b^Immunocompromised: solid organ transplant, bone marrow transplant, hematologic/oncologic malignancy, HIV.

Twenty clinically significant drug-associated OPAT-AEs (2.8%) were identified. Only 9 (1.3%) were recognized as abnormal via routine laboratory monitoring, after a median (IQR) of 26 (19–34) days, with only 3 resulting in unplanned health care utilization. Descriptions of the abnormal lab–associated OPAT-AEs are listed in Table 2. The overall incidence of laboratory monitoring–detected clinically significant OPAT-AEs was 2.7 per 1000 sets of weekly labs. We also found 34 (5%) clinically significant catheter-associated OPAT-AEs, which developed after a median (IQR) of 16 (10–26.5) days. Additional secondary outcomes are listed in Table 2, and univariate and multivariate logistic regressions are listed in Supplementary Tables 1 and 2. We noted no statistically significant differences in antibiotic utilized, duration of therapy, or OPAT modality (eg, home health care, skilled nursing facility, etc.) between the groups with or without clinically significant drug-associated OPAT-AEs. However, in the multivariate analysis, patients receiving OPAT via hemodialysis lines were 94% less likely (*P* = .011) and patients receiving OPAT via peripherally inserted central catheter/central venous catheters (PICC/CVCs) were 77% less likely (*P* < .0001) to have a catheter-associated OPAT-AE in comparison with patients with midlines. Younger age was also associated with clinically significant OPAT-AEs (*P* = .0042).

**Table 2.** Primary and Secondary Outcomes

Outcomes (n = 708)^a^	No. (%)
Clinically significant OPAT-related adverse events	55 (7.8)
Catheter-associated	35 (5)
Drug-associated	20 (2.8)
Abnormal lab	9 (1.3)
Thrombocytopenia	4 (0.6)
Acute kidney injury^b^	2 (0.3)
Elevated liver function tests^b^	2 (0.3)
Hypernatremia	1 (0.1)
Allergy/itching/rash	8 (1.1)
*Clostridioides difficile* infection	3 (0.4)
Time from start of therapy to OPAT-related adverse event, median (IQR), d	17 (10–27)
Catheter-associated adverse event, median (IQR), d	16 (10–26.5)
Drug-associated adverse event, median (IQR), d	20 (11.5–27.25)
Abnormal lab–associated adverse event, median (IQR), d	26 (19–34)
All-cause emergency department visit on treatment	100 (14.1)
All-cause readmission on treatment	88 (12.4)
All-cause readmission at 30 d	107 (16.2)
Infection-related readmission at 30 d	31 (4.7)
OPAT documentation^c^	…
Obtained initially missing labs	263
Clarified/adjusted/ordered labs	66
Triaged lab results^d^	32

Abbreviations: HD, hemodialysis; IQR, interquartile range; OPAT, outpatient parenteral antimicrobial therapy; PICC/CVC, peripherally inserted central catheter/central venous catheter; PIV, peripheral intravenous catheter; SCr, serum creatinine; ULN, upper limit normal.

^a^Six hundred sixty-six unique patients, 38 patients with >1 OPAT course of therapy.

^b^Patients were included in the analysis based on clinical significance of their adverse drug events, but also met these standard definitions for the listed adverse drug events: acute kidney injury: SCr increase of ≥0.3 mg/dL or >1.5 times baseline SCr; elevated liver function tests: 3 times ULN.

^c^OPAT documentation represents the total number of interventions in this cohort. Note that some courses of therapy required multiple interventions.

^d^OPAT nurse sent abnormal labs to OPAT pharmacist and prescriber to discuss.

## DISCUSSION

Previous studies assessing OPAT-AEs provided pooled analyses of various antimicrobials, including aminoglycosides and vancomycin, which are known to have less favorable safety profiles than beta-lactams [5–7, 9, 11]. To our knowledge, this is the first study specifically assessing the incidence of clinically significant OPAT-AEs for cefazolin and ceftriaxone, 2 of the most commonly used agents in OPAT. Across >700 courses of such therapy given for a median 30 days, our abnormal lab–associated OPAT-AE rate was only 1.3%. Furthermore, over half of the drug-associated OPAT-AEs were not identifiable via routine laboratory monitoring. Thus, our findings suggest that weekly laboratory monitoring for OPAT therapy with cefazolin and ceftriaxone may be excessive, with ∼370 sets of weekly labs having been needed to detect 1 OPAT-AE requiring a change in antimicrobial therapy.

The strengths of this study include the standardization of a clinically significant definition for drug-associated OPAT-AEs and the high percentage of weekly lab results obtained and reviewed within 72 hours of lab draw, rendering it unlikely that clinically significant abnormal lab–associated OPAT-AEs went undetected. The 1.3% incidence of abnormal lab–associated OPAT-AEs we found likely reflects the true incidence of OPAT-AEs for cefazolin and ceftriaxone. Conversely, we want to highlight the high burden on OPAT resource utilization that weekly laboratory monitoring entails. Our OPAT team documented 263 interventions to obtain initially missing labs and 66 interventions to clarify, adjust, or order labs to ensure guideline-recommended monitoring. We estimate that at least 25 OPAT team hours were devoted to obtaining initially missing laboratory monitoring for these 2 therapies, for uncertain benefit. Other potentially needless expenditures included the nursing time spent in obtaining and delivering these labs and the inconvenience, cost, iatrogenic anemia, and discomfort inflicted on patients. While we recognize that certain laboratory monitoring may allow for earlier identification of non-infection-related clinical problems, our experience indicates that it is likely to be a rare event.

Our results confirm previous studies regarding time to abnormal lab–associated OPAT-AEs [8, 10, 11]. Lam et al. reported changing their institutional laboratory monitoring policy for patients receiving nonvancomycin OPAT to 1 set of labs at week 3 of therapy because they found that this corresponded to the highest-risk period for delayed-onset hypersensitivity reactions [11]. However, this study specifically assessed neutropenia and not other potential abnormal lab–associated OPAT-AEs. Additionally, Frisby et al. confirmed that an OPAT duration of 2–4 weeks was associated with lower risk of OPAT-related complications [10]. However, they included antimicrobials with known higher incidence of AEs in comparison with beta-lactams. In our cohort, abnormal lab–associated OPAT-AEs occurred at a median (IQR) of 26 (19–34) days, which confirms that laboratory monitoring may not be necessary until patients have received at least 2 to 3 weeks of cefazolin or ceftriaxone.

The limitations of our study include its retrospective, single-institution design and our presumption of causation in deeming clinically significant AEs related to the patient's OPAT antimicrobials. However, each OPAT-AE was independently adjudicated by study investigators as reasonably having causation with the intravenous antimicrobial. For additional transparency, Supplementary Table 3 details cases of OPAT-AEs in patients also receiving concomitant oral antimicrobial therapy. Finally, due to the low number of clinically significant drug-associated OPAT-AEs, our multivariate modeling was not powered to capture true associations for this subgroup, so this exploratory analysis was only performed for all clinically significant OPAT-AEs. Results from this analysis were significant for increased risk with the use of midline catheters, concordant with previous literature associating midlines with higher rates of superficial venous thrombosis [12]. This is also concordant with the earlier time to catheter-associated AEs we identified, compared with both time to drug- or abnormal lab–associated AEs. Younger age was also associated in the multivariate analysis with increased risk for clinically significant OPAT-AEs, with a 2% increase in risk for every 1-year decrease in age, the clinical significance of which is likely marginal. Furthermore, we propose the likelihood of an interaction between younger age and the other variables identified in this analysis. The younger cohort subpopulation was more likely to receive OPAT in a home health care setting, and a greater percentage also received their antimicrobials via midlines (Supplementary Table 4), which have more plausible clinical explanations for their association with AEs than age alone.

In summary, cefazolin and ceftriaxone are well tolerated during OPAT, with few OPAT-AEs (inclusive of both catheter- and drug-related) mainly occurring beyond 3 weeks of therapy. As we identified just 2.7 clinically significant OPAT-AEs per 1000 sets of weekly labs, routine weekly laboratory monitoring for cefazolin and ceftriaxone in the OPAT setting may be excessive. Less intensive monitoring strategies than IDSA guidelines currently recommend are likely safe and should be explored.

## Supplementary Material

ofad606_Supplementary_Data

## Supplementary Data

Supplementary materials are available at *Open Forum Infectious Diseases* online. Consisting of data provided by the authors to benefit the reader, the posted materials are not copyedited and are the sole responsibility of the authors, so questions or comments should be addressed to the corresponding author.
