# Supplementary material for: Serious Adverse Events and Laboratory Monitoring Regimens for Outpatient Parenteral Antimicrobial Therapy With Cefazolin and Ceftriaxone
Source: Open Forum Infect Dis. 2023 Dec 2;10(12):ofad606. doi: 10.1093/ofid/ofad606 (PMC10727193; doi:10.1093/ofid/ofad606)
Supplement: ofad606_Supplementary_Data [file ofad606_supplementary_data.docx]

**Supplemental Table 1: Univariate Analysis of Patients Developing a Clinically Significant Adverse Event**

| **Univariate Analysis** | **No Drug-Associated Adverse Event**  **(N=688)** | **Any Drug-Associated Adverse Event**  **(N=20)** | **P-value** |
| --- | --- | --- | --- |
| Antimicrobial Agent |  |  | 1 |
| Cefazolin | 356 (51.7%) | 10 (50%) |  |
| Ceftriaxone | 332 (48.3%) | 10 (50%) |  |
| Age (years), mean + SD | 59.2 + 16.3 | 54.5 + 14.4 | 0.16 |
| Charlson Comorbidity Index score, median (IQR) | 5 (2, 7) | 3 (2, 6.5) | 0.24 |
| Immunocompromised^a^ | 185 (26.9%) | 6 (30%) | 0.79 |
| OPAT Modality |  |  | 0.13 |
| Home health care | 399 (58%) | 16 (80%) |  |
| Skilled nursing facility | 135 (19.6%) | 0 (0%) |  |
| Long-term acute care | 55 (8%) | 2 (10%) |  |
| Hemodialysis center | 50 (7.3%) | 1 (5%) |  |
| Infusion center | 48 (7%) | 1 (5%) |  |
| Other | 1 (0.1%) | 0 (0%) |  |
| Total Duration of Antibiotic Therapy (days), median (IQR) | 30.5 (19, 42) | 39.5 (24, 43.5) | 0.39 |
| Days of Therapy on Drug of Interest, median (IQR) | 30 (19, 42) | 21.5 (13.5, 33) | 0.014 |

^a^Immunocompromised: solid organ transplant, bone marrow transplant, hematologic/oncologic malignancy, human immunodeficiency virus

Abbreviations: SD = standard deviation; IQR = interquartile range; OR = odds ratio; CI = confidence interval; PICC/CVC = peripherally inserted central catheter/central venous catheter

**Supplemental Table 2:** **Multivariate Analysis of Patients Developing a Clinically Significant Adverse Event**

| **Multivariate Analysis**  **(All Clinically Significant Adverse Events, N=****55)^a^** | **OR** | **95% CI** | **P-value** |
| --- | --- | --- | --- |
| Vascular Access |  |  |  |
| HD line vs Midline | 0.064 | 0.008, 0.54 | 0.011 |
| PICC/CVC vs Midline | 0.232 | 0.115, 0.468 | < 0.0001 |
| OPAT Modality |  |  |  |
| Home health care vs Other | 1.801 | 0.900, 3.601 | 0.096 |
| Age (years) | 0.976 | 0.96, 0.992 | 0.0042 |

^a^Multivariate analysis performed for all adverse events; unable to perform on only drug-associated adverse events due to sample size

Abbreviations: HD = hemodialysis, PICC/CVC = peripherally inserted central catheter/central venous catheter

**Supplemental Table 3: Subgroup of Adverse Drug Events in Patients with Concomitant Oral Antimicrobial Therapy**

| **Concomitant Oral Antimicrobial Therapy (N=111)** | **N (%)** |
| --- | --- |
| Clinically Significant OPAT-related Adverse Events | 10 (9%) |
| Catheter-Associated | 5 (4.5%) |
| Drug-Associated | 5 (4.5%) |
| Cytopenia |  |
| Cefazolin + rifampin | 1 (0.9%) |
| Ceftriaxone + doxycycline + metronidazole | 1 (0.9%) |
| Acute kidney injury^a^ |  |
| Cefazolin + rifampin | 1 (0.9%) |
| *Clostridioides difficile* Infection |  |
| Ceftriaxone + vancomycin | 1 (0.9%) |
| Rash |  |
| Ceftriaxone + metronidazole | 1 (0.9%) |

^a^Patients were included in the analysis based on clinical significance of their adverse drug events, but also met the standard definition for acute kidney injury: SCr increase of > 0.3 mg/dL or > 1.5 times baseline SCr

**Supplemental Table 4: Age Stratification of Patients Developing a Clinically Significant Adverse Event**

| **Age Group Stratification**  **(All Clinically Significant Adverse Events, N=55)** | **OPAT Modality (%)** | | **Vascular Access (%)** | | |
| --- | --- | --- | --- | --- | --- |
|  | **Home health care** | **Other** | **PICC/CVC** | **Midline** | **HD line** |
| < 40 years (N=11) | 10 (91%) | 1 (9%) | 7 (64%) | 4 (36%) | 0 |
| 40 - < 50 years (N=10) | 9 (90%) | 1 (10%) | 6 (60%) | 4 (40%) | 0 |
| 50 - < 60 years (N=15) | 13 (87%) | 2 (13%) | 12 (80%) | 2 (13%) | 1 (7%) |
| > 60 years (N=19) | 11 (58%) | 8 (42%) | 15 (79%) | 4 (21%) | 0 |

Abbreviations: HD = hemodialysis, PICC/CVC = peripherally inserted central catheter/central venous catheter
